# Single-inhaler triple therapy fluticasone furoate/umeclidinium/vilanterol versus fluticasone furoate/vilanterol and umeclidinium/vilanterol in patients with COPD: results on cardiovascular safety from the IMPACT trial

**DOI:** 10.1186/s12931-020-01398-w

**Published:** 2020-06-05

**Authors:** Nicola C. Day, Subramanya Kumar, Gerard Criner, Mark Dransfield, David M. G. Halpin, MeiLan K. Han, C. Elaine Jones, Morrys C. Kaisermann, Sally Kilbride, Peter Lange, David A. Lomas, Neil Martin, Fernando J. Martinez, Dave Singh, Robert Wise, David A. Lipson

**Affiliations:** 1 GlaxoSmithKline, Stockley Park West, 1-3 Ironbridge Road, Uxbridge, Middlesex, UB11 1BT UK; 2 Lewis Katz School of Medicine at Temple University, Philadelphia, PA USA; 3 Division of Pulmonary, Allergy, and Critical Care Medicine, Lung Health Center, University of Alabama at Birmingham, Birmingham, AL USA; 4 University of Exeter Medical School, College of Medicine and Health, Exeter, UK; 5 University of Michigan, Pulmonary & Critical Care, Ann Arbor, MI USA; 6 GlaxoSmithKline, Research Triangle Park, NC USA; 7 GlaxoSmithKline, Collegeville, PA USA; 8 Department of Public Health, Section of Epidemiology, University of Copenhagen, Copenhagen, Denmark; 9 Medical Department, Herlev and Gentofte Hospital, Herlev, Denmark; 10 UCL Respiratory, University College London, London, UK; 11 GlaxoSmithKline, Brentford, UK; 12 University of Leicester, Leicester, UK; 13 New York-Presbyterian Weill Cornell Medical Center, New York, NY USA; 14 University of Manchester, Manchester University NHS Foundation Trust, Manchester, UK; 15 Division of Pulmonary and Critical Care Medicine, Johns Hopkins Medicine, Baltimore, MD USA; 16 Perelman School of Medicine, University of Pennsylvania, Philadelphia, PA USA

**Keywords:** COPD, Triple therapy, LAMA/LABA, ICS/LABA, Cardiovascular safety

## Abstract

**Background:**

This analysis of the IMPACT study assessed the cardiovascular (CV) safety of single-inhaler triple therapy with fluticasone furoate/umeclidinium/vilanterol (FF/UMEC/VI) versus FF/VI and UMEC/VI dual therapy.

**Methods:**

IMPACT was a 52-week, randomized, double-blind, multicenter Phase III study comparing the efficacy and safety of FF/UMEC/VI 100/62.5/25 mcg with FF/VI 100/25 mcg or UMEC/VI 62.5/25 mcg in patients ≥40 years of age with symptomatic chronic obstructive pulmonary disease (COPD) and ≥1 moderate/severe exacerbation in the previous year. The inclusion criteria for the study were intentionally designed to permit the enrollment of patients with significant concurrent CV disease/risk. CV safety assessments included proportion of patients with and exposure-adjusted rates of on-treatment CV adverse events of special interest (CVAESI) and major adverse cardiac events (MACE), as well as time-to-first (TTF) CVAESI, and TTF CVAESI resulting in hospitalization/prolonged hospitalization or death.

**Results:**

Baseline CV risk factors were similar across treatment groups. Overall, 68% of patients (*n* = 7012) had ≥1 CV risk factor and 40% (*n* = 4127) had ≥2. At baseline, 29% of patients reported a current/past cardiac disorder and 58% reported a current/past vascular disorder. The proportion of patients with on-treatment CVAESI was 11% for both FF/UMEC/VI and UMEC/VI, and 10% for FF/VI. There was no statistical difference for FF/UMEC/VI versus FF/VI or UMEC/VI in TTF CVAESI (hazard ratio [HR]: 0.98, 95% confidence interval [CI]: 0.85, 1.11; *p* = 0.711 and HR: 0.92, 95% CI: 0.78, 1.08; *p* = 0.317, respectively) nor TTF CVAESI leading to hospitalization/prolonged hospitalization or death (HR: 1.19, 95% CI: 0.93, 1.51; *p* = 0.167 and HR: 0.96, 95% CI: 0.72, 1.27; *p* = 0.760, respectively). On-treatment MACE occurred in ≤3% of patients across treatment groups, with similar prevalence and rates between treatments.

**Conclusions:**

In a symptomatic COPD population with a history of exacerbations and a high rate of CV disease/risk, the proportion of patients with CVAESI and MACE was 10–11% and 1–3%, respectively, across treatment arms, and the risk of CVAESI was low and similar across treatment arms. There was no statistically significant increased CV risk associated with the use of FF/UMEC/VI versus FF/VI or UMEC/VI, and UMEC/VI versus FF/VI.

**Trial registration:**

NCT02164513 (GSK study number CTT116855).

## Background

Chronic obstructive pulmonary disease (COPD) is a common respiratory disease characterized by chronic airflow limitation and persistent respiratory symptoms [1]. COPD is associated with a substantial clinical burden [1], was the third leading cause of death in the world in 2016, and is expected to remain a leading cause of death worldwide in 2030 [2, 3]. The main goals of COPD pharmacological treatment are to reduce symptoms, improve health status and exercise tolerance, and to reduce the risk of exacerbation and mortality [1].

Most patients with COPD present with at least one chronic comorbidity [4]. Clinicians must therefore take into consideration the effect of therapeutic intervention on comorbid diseases to ensure appropriate disease management [1, 5]. Cardiovascular (CV) disease, including coronary artery disease, heart failure, and arrhythmias, is a common comorbidity of COPD [6]. Exacerbations in COPD symptoms are associated with elevated CV disease risk or worse outcomes especially in patients with concomitant COPD and CV disease [1, 6, 7]. Similarly, the presence of CV comorbidities in patients with COPD has been associated with worse outcomes [8].

COPD and CV disease share many risk factors, including older age and history of smoking, as well as similar pathophysiological mechanisms and exposures [6, 9]. Static and dynamic hyperinflation, which alter venous return and cardiac output, hypoxemia, and systemic inflammation may each lead to increased risk of adverse CV events/disease, which can then in turn exacerbate COPD symptoms [6, 9–12].

Inhaled corticosteroids (ICS), long-acting β_2_-agonists (LABA) and long-acting muscarinic antagonists (LAMA) are a mainstay of COPD treatment [1]. There are concerns that LAMA and LABA therapy may be associated with a higher risk of cardiovascular adverse events, depending on dosage and receptor specificity, by signaling through β-adrenergic receptors and inhibiting muscarinic receptors, which are present in lung and heart tissue [7, 13–15]. Some studies have suggested an increased risk of CV events in patients with COPD receiving bronchodilators, although the evidence remains controversial [7, 14, 16–18]. Recent studies have found no increased CV risk with the use of inhaled COPD therapies or during escalation from ICS/LABA to ICS/LAMA/LABA triple therapy, but have noted that additional data are needed in patients with higher CV risk [7, 19–21].

In the InforMing the Pathway of COPD Treatment (IMPACT) study, once-daily single-inhaler triple therapy with fluticasone furoate/umeclidinium/vilanterol (FF/UMEC/VI) reduced the rate of moderate/severe exacerbations and improved lung function and health-related quality of life compared with dual therapy with FF/VI or UMEC/VI in patients ≥40 years of age with symptomatic COPD and a history of exacerbations [22]. FF/UMEC/VI also significantly reduced the rate of hospitalized exacerbations and all-cause mortality versus UMEC/VI, with a reduction in respiratory death, CV death, and death associated with the patients’ underlying COPD when compared with UMEC/VI [22].

Unlike many previous clinical studies, the IMPACT trial had broad entry criteria and included patients with significant concurrent CV disease/risk [23]. This allowed the assessment of efficacy and CV safety of these inhaled COPD therapies in a population that is more representative of real-world clinical practice. The aim of this pre-specified analysis was to assess the CV safety of FF/UMEC/VI versus FF/VI and UMEC/VI in the intent-to-treat (ITT) population of the IMPACT trial.

## Methods

### Study design

The IMPACT trial (GSK Study CTT116855; NCT02164513) was a 52-week randomized, double-blind, multicenter Phase III study, which compared the efficacy and safety of once-daily single-inhaler triple therapy with FF/UMEC/VI 100/62.5/25 mcg with once-daily dual therapy with FF/VI 100/25 mcg or UMEC/VI 62.5/25 mcg. The study design has been published previously [22, 23]. Briefly, after a 2-week run-in period during which patients continued their existing COPD medications, patients were randomized 2:2:1 to FF/UMEC/VI 100/62.5/25 mcg, FF/VI 100/25 mcg or UMEC/VI 62.5/25 mcg, all administered once daily via the ELLIPTA dry powder inhaler.

### Study population

Inclusion/exclusion criteria of the IMPACT trial have been described previously [22]. Briefly, eligible patients were ≥40 years of age with symptomatic COPD (COPD Assessment Test score ≥10), and had a forced expiratory volume in 1 s (FEV_1_) < 50% of predicted normal values and a history of ≥1 moderate or severe exacerbation in the previous year, or a FEV_1_ 50–80% of predicted normal values and a history of ≥2 moderate or ≥1 severe exacerbations in the previous year [22]. Patients were excluded if they had unstable or life-threatening CV disease. However, patients with a history of previous myocardial infarction (MI; >6 months prior to screening), New York Heart Association (NYHA) Class 1–3 heart failure, and unstable or life-threatening cardiac arrhythmia requiring intervention (>3 months prior to Screening) were eligible to participate in the study [23]. Patients using ≤3 L/min of supplemental oxygen at rest at screening were also eligible to participate. The presence of CV risk factors was assessed at baseline, based on data captured in the electronic case report form (eCRF). Patients with ≥1 of the following past or current medical conditions were classed as having a CV risk factor: angina pectoris, coronary artery disease, MI, arrhythmia, congestive heart failure, hypertension, cerebrovascular accident, carotid or aorto-femoral vascular disease, diabetes mellitus, and hypercholesterolemia.

### Study endpoints

Safety endpoints investigated in this analysis included the incidence of investigator-reported adverse events (AEs) of special interest (AESI), and the incidence of major adverse cardiovascular events (MACE). AESI were pre-specified groups of AEs of special interest for FF, UMEC, or VI, or for patients with COPD, allowing for a comprehensive review of safety data that is not limited to a specific AE Preferred Term. For the AESI of Cardiovascular effects (termed CVAESI here), Standardized Medical Dictionary for Regulatory Activities (MedDRA) Queries (SMQs) were used. MedDRA SMQs are validated, pre-determined sets of MedDRA Preferred Terms grouped together to facilitate the capture all plausible events linked to a disease process [24]. In the IMPACT study, CVAESI included cardiac arrhythmia (contains selected sub-SMQs), cardiac failure (SMQ), ischemic heart disease (SMQ), hypertension (SMQ), and central nervous system (CNS) hemorrhages and cerebrovascular conditions (SMQ). The CVAESI SMQs and constituent Preferred Terms that were reported in the IMPACT study are provided in **Supplementary Table** **1**. Serious CVAESI were those CVAESI reported as serious AEs (SAEs; as specified in the protocol [23]). CVAESI resulting in hospitalization/prolonged hospitalization or death – referred to herein as ‘hospitalized or fatal CVAESI’ – were classified as such according to investigator-reported information in the eCRF.

MACE was determined from independently adjudicated CV deaths and investigator-reported non-fatal AEs and was broadly and narrowly defined. Broad MACE included adjudicated CV deaths, non-fatal CNS hemorrhages and cerebrovascular conditions (SMQ), non-fatal MI (SMQ) and non-fatal other ischemic heart disease (SMQ). Narrow MACE included adjudicated CV deaths, non-fatal CNS hemorrhages and cerebrovascular conditions (SMQ), non-fatal MI Preferred Term and acute MI Preferred Term.

On-treatment CV safety and MACE were assessed as pre-specified analyses in the IMPACT study. On-treatment CV safety assessments included: (1) the proportion of patients with and exposure-adjusted rate of CVAESI and serious CVAESI; (2) risk (time-to-first [TTF]) of CVAESI; (3) risk (TTF) of hospitalized or fatal CVAESI (overall and by baseline CV risk factors); and (4) the proportion of patients with and exposure-adjusted rate of MACE.

### Statistical analyses

The proportion of patients with on-treatment MACE was reported as a percentage and exposure-adjusted rate per 1000-patient years. The risk of on-treatment CVAESI and on-treatment hospitalized or fatal CVAESI was evaluated using a TTF event analysis and derived using a Cox proportional hazards model with covariates of treatment group and geographical region.

## Results

### Patients

The ITT population included a total of 10,355 patients (FF/UMEC/VI: *N* = 4151, FF/VI: *N* = 4134, UMEC/VI: *N* = 2070) [22]. Baseline characteristics, CV disorders, and risk factors were similar across treatment groups (Table 1). At baseline, 29% (*n* = 2964) of patients reported a current or past cardiac disorder (coronary artery disease *n* = 1252 [12%]; arrhythmia *n* = 816 [8%]; angina pectoris *n* = 737 [7%]; MI *n* = 681 [7%]; congestive heart failure *n* = 539 [5%]). Overall, 58% (*n* = 6021) of patients reported a current or past vascular disorder (hypertension *n* = 5446 [53%]; cerebrovascular accident *n* = 458 [4%]; carotid or aorto-femoral vascular disease *n* = 342 [3%]).

**Table 1:** Baseline characteristics (ITT population)

	FF/UMEC/VI *N* = 4151	FF/VI *N* = 4134	UMEC/VI *N* = 2070	Overall *N* = 10,355
**Age, mean (SD), years**	65.3 (8.2)	65.3 (8.3)	65.2 (8.3)	65.3 (8.3)
**Gender, male, n (%)**	2766 (67)	2748 (66)	1356 (66)	6870 (66)
**BMI, mean (SD), kg/m**^**2**^	26.6 (6.2)	26.7 (6.1)	26.6 (5.9)	26.6 (6.1)
**Smoking status, n (%)**				
Current smoker	1436 (35)	1423 (34)	728 (35)	3587 (35)
Former smoker	2715 (65)	2711 (66)	1342 (65)	6768 (65)
**Moderate or severe COPD exacerbations in previous year, n (%)**				
0	2 (< 1)	5 (< 1)	2 (< 1)	9 (< 1)
1	1853 (45)	1907 (46)	931 (45)	4691 (45)
2	1829 (44)	1768 (43)	890 (43)	4487 (43)
≥3	467 (11)	454 (11)	247 (12)	1168 (11)
**Post-bronchodilator FEV**_**1**_**% predicted, mean (SD)**	47.5 (15.0)	45.5 (14.8)	45.4 (14.7)	45.5 (14.8)
**Current/past Cardiac disorders**^a^**, n (%)**	1194 (29)	1173 (28)	597 (29)	2964 (29)
**Current/past Vascular disorders**^a^**, n (%)**	2362 (57)	2438 (59)	1221 (59)	6021 (58)
**CV risk factors**^a^**, n (%)**				
0	1365 (33)	1322 (32)	656 (32)	3343 (32)
1	1147 (28)	1158 (28)	580 (28)	2885 (28)
≥1	2786 (67)	2812 (68)	1414 (68)	7012 (68)
≥2	1639 (39)	1654 (40)	834 (40)	4127 (40)
**CV risk factors**^a^**, n (%)**				
Hypertension	2132 (51)	2207 (53)	1107 (53)	5446 (53)
Hypercholesterolemia	1354 (33)	1332 (32)	681 (33)	3367 (33)
Diabetes mellitus	641 (15)	645 (16)	313 (15)	1599 (15)
Coronary artery disease	510 (12)	488 (12)	254 (12)	1252 (12)
Arrhythmia	335 (8)	323 (8)	158 (8)	816 (8)
Angina pectoris	291 (7)	307 (7)	139 (7)	737 (7)
Myocardial infarction	270 (7)	274 (7)	137 (7)	681 (7)
Congestive heart failure	223 (5)	192 (5)	124 (6)	539 (5)
Cerebrovascular accident	199 (5)	165 (4)	94 (5)	458 (4)
Vascular disease^b^	133 (3)	148 (4)	61 (3)	342 (3)
**Family history of CV risk factors**^a^**, n (%)**^c^				
Premature coronary artery disease^d^				
Yes	432 (10)	430 (10)	234 (11)	1096 (11)
No	3160 (76)	3097 (75)	1529 (74)	7786 (75)
Unknown	559 (13)	607 (15)	307 (15)	1473 (14)
Myocardial infarction				
Yes	651 (16)	673 (16)	361 (17)	1685 (16)
No	3004 (72)	2954 (71)	1431 (69)	7389 (71)
Unknown	496 (12)	507 (12)	278 (13)	1281 (12)
Stroke				
Yes	448 (11)	464 (11)	230 (11)	1142 (11)
No	3191 (77)	3150 (76)	1565 (76)	7906 (76)
Unknown	512 (12)	520 (13)	275 (13)	1307 (13)

^a^As captured in the electronic case report form; ^b^carotid or aorto-femoral vascular disease; ^c^history in first degree relatives only; ^d^women < 65 years-old, men < 55 years-old.

*BMI* Body mass index; *COPD* Chronic obstructive pulmonary disease; *CV* Cardiovascular; *FEV*_*1*_ Forced expiratory volume in 1 s; *FF* Fluticasone furoate; *ITT* Intent-to-treat; *SD* Standard deviation; *UMEC* Umeclidinium; *VI* Vilanterol

Overall, 68% (*n* = 7012) of patients had at least one CV risk factor and 40% (*n* = 4127) had at least 2 (Table 1). The reported frequency of risk factors was consistent across treatment groups. The CV risk factors most frequently reported (≥10% of patients) were hypertension (53%), hypercholesterolemia (33%), diabetes mellitus (15%), and coronary artery disease (12%) (Table 1).

### On-treatment CVAESI (defined by MedDRA SMQ [see Supplementary Table 1])

The most frequently reported on-treatment AESI in the ITT population was Cardiovascular Effects [22], referred to in the current paper as CVAESI. The proportion of patients with and exposure-adjusted rates of on-treatment CVAESI were similar across all treatment groups (proportion [rate per 1000 patient-years]: 11% [167.2], 10% [157.0], and 11% [166.6] for FF/UMEC/VI, FF/VI, and UMEC/VI, respectively) (Table 2). Cardiac arrhythmia (comprised of sub-SMQs; **Supplementary Table** **1**) was reported most frequently and occurred in a similar proportion of patients (4% in all treatment groups) and with similar adjusted exposure rates across treatment groups, followed by Cardiac failure (SMQ) and Hypertension (SMQ), both of which occurred in 3% of patients in all treatment groups (Table 2). Ischemic heart disease (SMQ) occurred in 1–2% of patients across treatment groups, with exposure-adjusted rates for FF/UMEC/VI, FF/VI and UMEC/VI of 26.1, 18.5, and 30.6 per 1000 patient-years, respectively (Table 2). The proportion of patients with and rate of CVAESI (including sub-SMQs) by baseline CV risk factors were generally similar between treatment arms, with no pattern of events observed (**Supplementary Table****2**).

**Table 2:** Summary of on-treatment CVAESI by SMQs and sub-SMQs (ITT population)

Special interest group/subgroup	FF/UMEC/VI (***N*** = 4151)		FF/VI (***N*** = 4134)		UMEC/VI (***N*** = 2070)	
**Total duration at risk (patient-years)**	3714.9		3457.9		1698.3	
	**n (%)**	**Rate [#]**	**n (%)**	**Rate [#]**	**n (%)**	**Rate [#]**
**CVAESI**^a^	**450 (11)**	**167.2 [621]**	**430 (10)**	**157.0 [543]**	**224 (11)**	**166.6 [283]**
Cardiac arrhythmia	153 (4)	50.9 [189]	161 (4)	51.5 [178]	81 (4)	51.2 [87]
Arrhythmia-related investigations, signs and symptoms (SMQ)	63 (2)	19.7 [73]	71 (2)	22.8 [79]	33 (2)	20.6 [35]
Bradyarrhythmia terms, nonspecific (SMQ)	0 (0)	0 [0]	0 (0)	0 [0]	0 (0)	0 [0]
Cardiac arrhythmia terms, nonspecific (SMQ)	7 (< 1)	1.9 [7]	10 (< 1)	2.9 [10]	6 (< 1)	3.5 [6]
Conduction defects (SMQ)	20 (< 1)	5.7 [21]	16 (< 1)	4.6 [16]	10 (< 1)	5.9 [10]
Disorders of sinus node function (SMQ)	3 (< 1)	0.8 [3]	2 (< 1)	0.6 [2]	0 (0)	0 [0]
Supraventricular tachyarrhythmias (SMQ)	65 (2)	18.8 [70]	51 (1)	15.9 [55]	27 (1)	16.5 [28]
Tachyarrhythmia terms, nonspecific (SMQ)	3 (< 1)	0.8 [3]	4 (< 1)	1.2 [4]	1 (< 1)	0.6 [1]
Ventricular tachyarrhythmias (SMQ)	13 (< 1)	3.5 [13]	13 (< 1)	3.8 [13]	7 (< 1)	4.1 [7]
Cardiac failure (SMQ)	138 (3)	42.5 [158]	126 (3)	42.8 [148]	68 (3)	44.8 [76]
CNS hemorrhages and cerebrovascular conditions (SMQ)	41 (< 1)	12.1 [45]	28 (< 1)	9.3 [32]	11 (< 1)	6.5 [11]
Hypertension (SMQ)	113 (3)	35.5 [132]	115 (3)	35.0 [121]	54 (3)	34.2 [58]
Ischemic heart disease (SMQ)	80 (2)	26.1 [97]	57 (1)	18.5 [64]	47 (2)	30.6 [52]

#, number of events. Rates are reported as number of events per 1000 patient-years, calculated as the number of events × 1000, divided by the total duration at risk. ^a^Note, a patient may have experienced more than one CVAESI

*CNS* Central nervous system; *CVAESI*, Cardiovascular adverse event of special interest; *FF* Fluticasone furoate; *ITT* Intent-to-treat; *MedDRA* Medical Dictionary for Regulatory Activities; *n* Number of patients; *SMQ* Standardized MedDRA Query; *UMEC* Umeclidinium; *VI* Vilanterol

On-treatment serious CVAESI occurred in 3–4% of patients across treatment arms (Table 3). On-treatment fatal serious CVAESI were reported in <1% of patients in each treatment group, with exposure-adjusted rates (per 1000 patient-years) of 7.0 for FF/UMEC/VI, 6.9 for FF/VI, and 11.2 for UMEC/VI group.

**Table 3:** Summary of on-treatment serious and fatal serious CVAESI^a^ by SMQs and sub-SMQs (ITT population)

Special interest group/subgroup	FF/UMEC/VI (***N*** = 4151)		FF/VI (***N*** = 4134)		UMEC/VI (***N*** = 2070)	
**Total duration at risk (patient-years)**	3714.9		3457.9		1698.3	
	**n (%)**	**Rate [#]**	**n (%)**	**Rate [#]**	**n (%)**	**Rate [#]**
**Serious CVAESI**^b^	**151 (4)**	**54.1 [201]**	**119 (3)**	**38.2 [132]**	**74 (4)**	**51.2 [87]**
Cardiac arrhythmia	47 (1)	15.1 [56]	40 (< 1)	11.9 [41]	27 (1)	17.1 [29]
Arrhythmia-related investigations, signs and symptoms (SMQ)	22 (< 1)	5.9 [22]	17 (< 1)	5.2 [18]	14 (< 1)	8.2 [14]
Bradyarrhythmia terms, nonspecific (SMQ)	0 (0)	0 [0]	0 (0)	0 [0]	0 (0)	0 [0]
Cardiac arrhythmia terms, nonspecific (SMQ)	0 (0)	0 [0]	0 (0)	0 [0]	0 (0)	0 [0]
Conduction defects (SMQ)	1 (< 1)	0.3 [1]	1 (< 1)	0.3 [1]	3 (< 1)	1.8 [3]
Disorders of sinus node function (SMQ)	1 (< 1)	0.3 [1]	1 (< 1)	0.3 [1]	0 (0)	0 [0]
Supraventricular tachyarrhythmias (SMQ)	26 (< 1)	7.8 [29]	16 (< 1)	4.6 [16]	9 (< 1)	5.3 [9]
Tachyarrhythmia terms, nonspecific (SMQ)	1 (< 1)	0.3 [1]	0 (0)	0 [0]	1 (< 1)	0.6 [1]
Ventricular tachyarrhythmias (SMQ)	2 (< 1)	0.5 [2]	5 (< 1)	1.4 [5]	2 (< 1)	1.2 [2]
Cardiac failure (SMQ)	45 (1)	14.8 [55]	33 (< 1)	9.8 [34]	15 (< 1)	10.6 [18]
CNS hemorrhages and cerebrovascular conditions (SMQ)	32 (< 1)	9.4 [35]	20 (< 1)	6.1 [21]	7 (< 1)	4.1 [7]
Hypertension (SMQ)	6 (< 1)	1.6 [6]	4 (< 1)	1.2 [4]	2 (< 1)	1.2 [2]
Ischemic heart disease (SMQ)	44 (1)	13.2 [49]	32 (< 1)	9.3 [32]	29 (1)	18.3 [31]
**Fatal serious CVAESI**^b^	**21 (< 1)**	**7.0 [26]**	**24 (< 1)**	**6.9 [24]**	**19 (< 1)**	**11.2 [19]**
Cardiac arrhythmia	12 (< 1)	3.8 [4]	9 (< 1)	2.6 [9]	11 (< 1)	6.5 [11]
Arrhythmia-related investigations, signs and symptoms (SMQ)	11 (< 1)	3.0 [11]	8 (< 1)	2.3 [8]	10 (< 1)	5.9 [10]
Bradyarrhythmia terms, nonspecific (SMQ)	0 (0)	0 [0]	0 (0)	0 [0]	0 (0)	0 [0]
Cardiac arrhythmia terms, nonspecific (SMQ)	0 (0)	0 [0]	0 (0)	0 [0]	0 (0)	0 [0]
Conduction defects (SMQ)	0 (0)	0 [0]	0 (0)	0 [0]	0 (0)	0 [0]
Disorders of sinus node function (SMQ)	0 (0)	0 [0]	0 (0)	0 [0]	0 (0)	0 [0]
Supraventricular tachyarrhythmias (SMQ)	2 (< 1)	0.5 [2]	0 (0)	0 [0]	1 (< 1)	0.6 [1]
Tachyarrhythmia terms, nonspecific (SMQ)	0 (0)	0 [0]	0 (0)	0 [0]	0 (0)	0 [0]
Ventricular tachyarrhythmias (SMQ)	2 (< 1)	0.5 [2]	0 (0)	0 [0]	1 (< 1)	0.6 [1]
Cardiac failure (SMQ)	4 (< 1)	1.1 [4]	6 (< 1)	1.7 [6]	3 (< 1)	1.8 [3]
CNS hemorrhages and cerebrovascular conditions (SMQ)	3 (< 1)	1.3 [5]	7 (< 1)	2.0 [7]	1 (< 1)	0.6 [1]
Hypertension (SMQ)	0 (0)	0 [0]	0 (0)	0 [0]	0 (0)	0 [0]
Ischemic heart disease (SMQ)	3 (< 1)	0.8 [3]	2 (< 1)	0.6 [2]	4 (< 1)	2.4 [4]

^a^Serious as specified in the study protocol [23]; ^b^Note, a patient may have experienced more than one CVAESI (including those that led to a fatal outcome). #, number of events. Rates are reported as number of events per 1000 patient-years, calculated as the number of events × 1000, divided by the total duration at risk

*CNS* Central nervous system; *CVAESI* Cardiovascular adverse event of special interest; *FF* Fluticasone furoate; *ITT* Intent-to-treat; *MedDRA* Medical Dictionary for Regulatory Activities; *n* Number of patients; *SMQ* Standardized MedDRA Query; *UMEC* Umeclidinium; *VI* Vilanterol

### Risk (TTF analysis) of on-treatment CVAESI

**Fig. 1:**
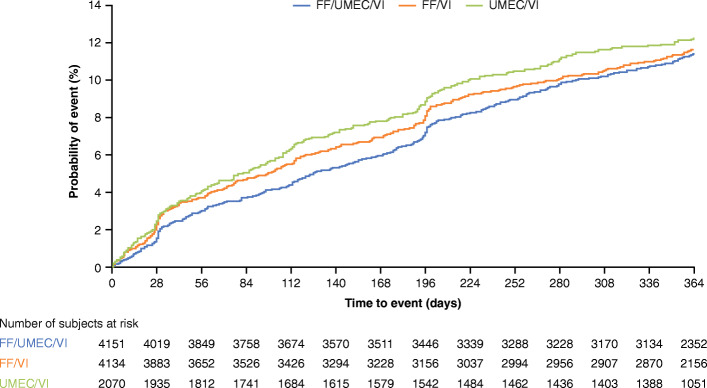
Kaplan–Meier plot of TTF on-treatment CVAESI. CVAESI, cardiovascular adverse event of special interest; FF, fluticasone furoate; TTF, time to first; UMEC, umeclidinium; VI, vilanterol

**Fig. 2:**
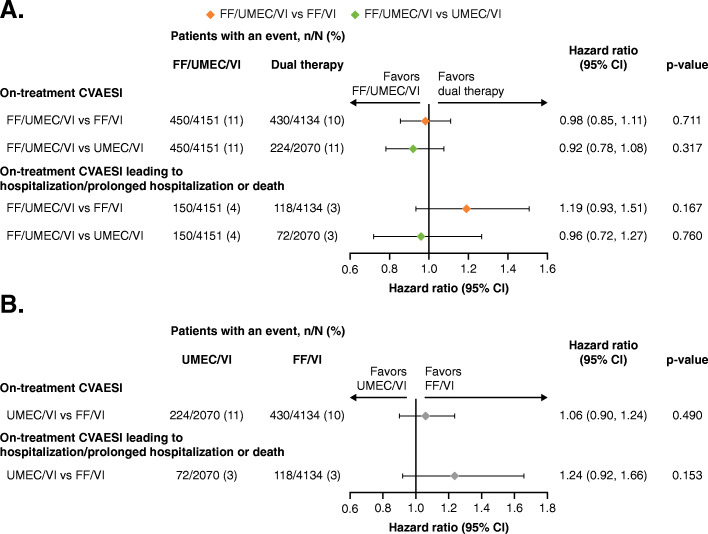
TTF on-treatment CVAESI and hospitalized or fatal CVAESI. **a**. FF/UMEC/VI versus FF/VI and UMEC/VI. **b**. UMEC/VI versus FF/VI. Hospitalized or fatal CVAESI refers to any CVAESI that resulted in hospitalization/prolonged hospitalization or death. CI, confidence interval; CVAESI, cardiovascular adverse event of special interest; FF, fluticasone furoate; n, number of patients with an event; N, number of patients in subgroup; TTF, time to first; UMEC, umeclidinium; VI, vilanterol

Based on an analysis of TTF event, the risk of experiencing a CVAESI was similar for FF/UMEC/VI compared with FF/VI (hazard ratio [HR]: 0.98, 95% CI: 0.85, 1.11; *p* = 0.711), FF/UMEC/VI compared with UMEC/VI (HR: 0.92, 95% CI: 0.78, 1.08; *p* = 0.317) and UMEC/VI compared with FF/VI (HR: 1.06, 95% CI: 0.90, 1.24; *p* = 0.490) (Figs 1 and 2).

**Fig. 3:**
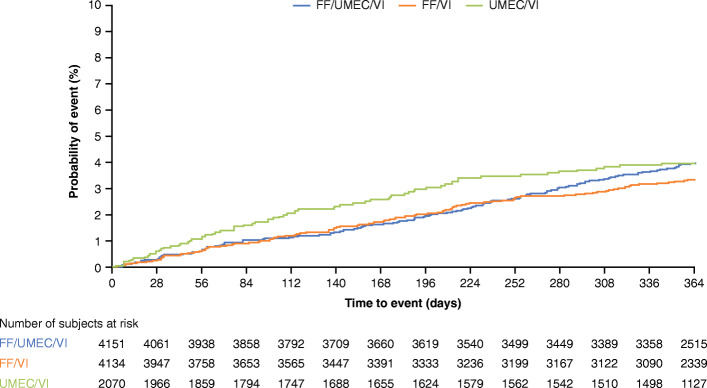
Kaplan–Meier plot of TTF on-treatment hospitalized or fatal CVAESI. Hospitalized or fatal CVAESI refers to any CVAESI that resulted in hospitalization/prolonged hospitalization or death. CVAESI, cardiovascular adverse event of special interest; FF, fluticasone furoate; HR, hazard ratio; TTF, time to first; UMEC, umeclidinium; VI, vilanterol

The proportion of patients with hospitalized or fatal CVAESI was 4, 3, and 3% for FF/UMEC/VI, FF/VI, and UMEC/VI, respectively (Fig. 2a). There were no statistically significant differences in risk of hospitalized or fatal CVAESI for FF/UMEC/VI versus FF/VI (HR: 1.19, 95% CI: 0.93, 1.51; *p* = 0.167) or UMEC/VI (HR: 0.96, 95% CI: 0.72, 1.27; *p* = 0.760), and UMEC/VI versus FF/VI (HR: 1.24, 95% CI: 0.92, 1.66; *p* = 0.153) in the overall ITT population (Figs. 2 and 3). A numerical increased risk of hospitalized or fatal CVAESI was seen with FF/UMEC/VI and UMEC/VI compared with FF/VI (Fig. 2).

**Fig. 4:**
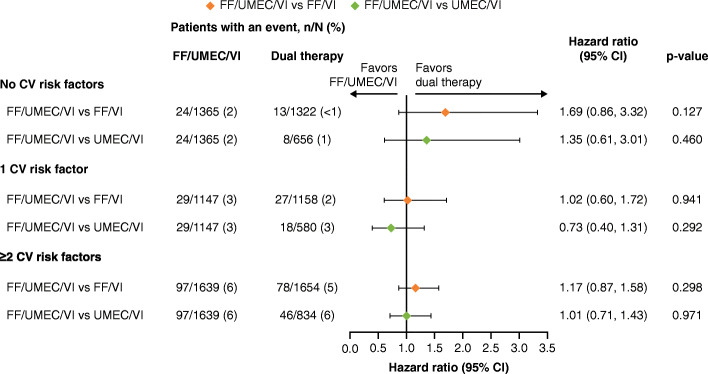
TTF on-treatment hospitalized or fatal CVAESI according to CV risk factors*. *In ≥3% of patients in any treatment group. Hospitalized or fatal CVAESI refers to any CVAESI that resulted in hospitalization/prolonged hospitalization or death. CI, confidence interval; CV, cardiovascular; CVAESI, cardiovascular adverse event of special interest; FF, fluticasone furoate; n, number of patients with an event; N, number of patients in subgroup; TTF, time to first; UMEC, umeclidinium; VI, vilanterol

There were no statistically significant differences in the risk of hospitalized or fatal CVAESI between the treatment groups when assessed by CV risk factor subgroup (Fig. 4), although a numerical increased risk of hospitalized or fatal CVAESI with FF/UMEC/VI compared with FF/VI was seen in patients who had no CV risk factors at baseline.

### Prevalence and rates of on-treatment MACE

The proportion of patients with and exposure-adjusted rates for any on-treatment MACE using the broad and narrow definitions were similar across treatment groups, with no consistent pattern seen between individual MACE categories (Table 4). The proportion of patients with MACE using the narrow definition was 2, 1, and 2% for FF/UMEC/VI, FF/VI, and UMEC/VI, respectively, with exposure-adjusted rates of 22.3, 18.8, and 22.4 per 1000 patient-years (Table 4). The proportion of patients with MACE using the broad definition was 3, 2, and 3% for FF/UMEC/VI, FF/VI, and UMEC/VI, respectively. The broad MACE exposure-adjusted rate was 44.7, 35.3, and 44.8 per 1000 patient-years for FF/UMEC/VI, FF/VI, and UMEC/VI, respectively (Table 4). The proportion of patients with adjudicated CV deaths was low across all treatment groups (<1%), with numerically lower exposure-adjusted rates observed in the FF/UMEC/VI and FF/VI groups (5.4 and 7.8 respectively) compared with the UMEC/VI group (9.4) (Table 4).

**Table 4:** On-treatment MACE (ITT population)

	FF/UMEC/VI (***N*** = 4151)		FF/VI (***N*** = 4134)		UMEC/VI (***N*** = 2070)	
**Total duration at risk (patient-years)**	3714.9		3457.9		1698.3	
	**n (%)**	**Rate [#]**	**n (%)**	**Rate [#]**	**n (%)**	**Rate [#]**
**Narrow definition**						
**Any MACE**	**80 (2)**	**22.3 [83]**	**60 (1)**	**18.8 [65]**	**37 (2)**	**22.4 [38]**
Adjudicated CV death	20 (< 1)	5.4 [20]	27 (< 1)	7.8 [27]	16 (< 1)	9.4 [16]
Non-fatal CNS hemorrhages and cerebrovascular conditions (SMQ)	38 (< 1)	10.8 [40]	21 (< 1)	7.2 [25]	10 (< 1)	5.9 [10]
Non-fatal MI (PT)	9 (< 1)	2.4 [9]	6 (< 1)	1.7 [6]	5 (< 1)	2.9 [5]
Non-fatal acute MI (PT)	13 (< 1)	3.8 [14]	7 (< 1)	2.0 [7]	7 (< 1)	4.1 [7]
**Broad definition**						
**Any MACE**	**133 (3)**	**44.7 [166]**	**100 (2)**	**35.3 [122]**	**66 (3)**	**44.8 [76]**
Adjudicated CV death	20 (< 1)	5.4 [20]	27 (< 1)	7.8 [27]	16 (< 1)	9.4 [16]
Non-fatal CNS hemorrhages and cerebrovascular conditions (SMQ)	38 (< 1)	10.8 [40]	21 (< 1)	7.2 [25]	10 (< 1)	5.9 [10]
Non-fatal MI (SMQ)	49 (1)	14.0 [52]	29 (< 1)	9.3 [32]	24 (1)	14.7 [25]
Non-fatal other ischemic heart disease (SMQ)	41 (< 1)	14.5 [54]	32 (< 1)	11.0 [38]	25 (1)	14.7 [25]

#, number of events. Rates are reported as number of events per 1000 patient-years, calculated as the number of events × 1000, divided by the total duration at risk

*CNS* Central nervous system; *CV* Cardiovascular; *FF* Fluticasone furoate; *ITT* Intent-to-treat; *MACE* Major adverse cardiac event; *MedDRA* Medical Dictionary for Regulatory Activities; *MI* Myocardial infarction; *n*, number of patients; *PT* Preferred Term; *SMQ* Standardized MedDRA Query; *UMEC* Umeclidinium; *VI* Vilanterol

## Discussion

IMPACT was a large trial in patients with symptomatic COPD and at risk of exacerbation evaluating the efficacy and safety of triple ICS/LAMA/LABA therapy versus dual LAMA/LABA or ICS/LABA therapy using the same molecules, doses and delivery device. The study had broad inclusion criteria, in particular with regards to significant concurrent CV disease/risk [22], compared with previously reported randomized controlled trials. In the IMPACT trial, patients with significant pre-existing CV disease were included and, therefore, the trial population is more likely to accurately reflect the real-world COPD population. At baseline, 68% of patients had at least one CV risk factor, and 40% had at least two. Approximately half of the patients across all treatment groups presented with vascular disorders at baseline and 16% had cardiac disorders.

This study shows that, in this symptomatic COPD population with a history of exacerbations including approximately two-thirds of patients with at least one CV risk factor at baseline, the proportion of patients with on-treatment CVAESI was 10–11% and with on-treatment MACE was 1–3%, without a consistent pattern across treatment groups. Furthermore, although the IMPACT study was not powered to assess CV safety, a low risk of on-treatment CVAESI was seen and there was no statistically significant increase in the risk of CVAESI, or hospitalized or fatal CVAESI, with FF/UMEC/VI versus either dual therapy; this was consistently observed irrespective of the number of baseline CV risk factors. There was a non-statistically significant increase in the risk of hospitalized or fatal CVAESI with FF/UMEC/VI and UMEC/VI compared with FF/VI. In addition, in patients who had no CV risk factors at baseline, there was a non-statistically significant increase in the risk of hospitalized or fatal CVAESI with FF/UMEC/VI compared with FF/VI.

Differences in the exposure-adjusted rates of on-treatment CVAESI and serious CVAESI, as well as narrow and broad MACE were small between the FF/UMEC/VI group and the FF/VI and UMEC/VI dual therapy groups. Any observed differences were likely due to the small number of events rather than an effect of the drug itself.

The exposure-adjusted rate of on-treatment CVAESI observed in the IMPACT trial should also be viewed in the context of using AESI for assessing CV safety outcomes. The use of CVAESI is a more conservative approach than using individual CV AE Preferred Terms or MACE, since the CVAESI encompasses a broad list of CV AE Preferred Terms that are pre-defined by the MedDRA. Furthermore, the IMPACT trial population reflects a population with a heavy CV risk factor burden when compared with the general COPD population as reported in a pooled analysis of the National Health and Nutrition Examination Surveys (NHANES) data [25], suggesting that the benefit seen with FF/UMEC/VI may extend outside the clinical trial environment.

Recent studies including meta-analyses and systematic reviews have demonstrated no increased CV risk during escalation from LAMA or LABA monotherapy to dual LAMA/LABA therapy, nor from ICS/LABA to ICS/LAMA/LABA triple therapy [7, 19, 20, 26, 27]; however, patients with high CV risk were not specifically included in some of these studies. The studies published so far have not highlighted CV safety concerns for UMEC [15], although, results are awaited from an ongoing observational study specifically investigating the effect of UMEC/VI versus tiotropium (TIO) on CV safety [28].

In a 2018 case-control study investigating dual LAMA/LABA therapy in more than 280,000 patients with COPD in Taiwan, a 1.5-fold increase of severe CV risk was demonstrated in patients who were naïve to LAMA/LABA treatment compared with patients who had prior exposure, regardless of exacerbation history or CV disease status [29]. However, this effect was only observed within 30 days of the onset of treatment; beyond 30 days of treatment the risk waned and subsequently reached lower than baseline levels [29]. As such, these results should be interpreted with caution, since if the increased risk of CV events was truly treatment-related the effect seen would be expected to continue beyond 30 days. This may reflect misdiagnosis, with cardiac symptoms being mistaken for COPD-related symptoms. Alternatively, it may suggest that this study was confounded by indication, as the study evaluated patients who newly initiated LAMA/LABA, i.e., likely to be symptomatic and requiring maximal bronchodilation due to the severity of their COPD; these patients would therefore be unstable and likely to be at a higher risk of experiencing adverse CV events [6].

Other clinical trials of single-inhaler triple therapy with ICS/LAMA/LABA have also shown similar CV safety profiles for triple therapy compared with LAMA monotherapy or ICS/LABA therapy. The TRILOGY (NCT01917331), TRINITY (NCT01911364), and TRIBUTE (NCT02579850) studies compared single-inhaler beclomethasone dipropionate/formoterol fumarate/glycopyrronium bromide triple therapy versus beclomethasone dipropionate/formoterol fumarate (ICS/LABA), TIO, and beclomethasone dipropionate/formoterol fumarate + TIO in multiple inhalers, and indacaterol/glycopyrronium (LABA/LAMA) over 52 weeks [30–32]. These studies showed similar prevalence of CV AEs and SAEs (e.g., ischemic heart disease and cardiac failure) and MACE between single-inhaler triple therapy and comparator arms [30–32]. However, these studies excluded patients with clinically significant CV disease, such as unstable ischemic heart disease, NYHA Class 3/4, left ventricular failure and acute MI, and patients with atrial fibrillation [30–32]. In contrast, the IMPACT study was designed with broader inclusion criteria with regards to CV disease and permitted participation of patients with history of previous MI (>6 months prior to screening), NYHA Class 1–3 heart failure, and unstable or life-threatening cardiac arrhythmia requiring intervention (>3 months prior to Screening) [22]. The IMPACT study results therefore support and expand the findings from other studies of triple therapies and those from recent meta-analyses and systematic reviews which demonstrated no increased CV risk with the use of inhaled COPD therapies [7, 19, 20]. These findings also support the overall favorable CV safety profile of FF/UMEC/VI triple therapy for the treatment of patients with symptomatic COPD and a history of exacerbations and are consistent with the extensive CV safety database for FF/VI, UMEC/VI, and UMEC monotherapy.

The data presented within this analysis, however, should be interpreted within the context of some potential limitations. Firstly, the study was not primarily designed or statistically powered for specifically assessing CV safety; secondly, the number of patients presenting with CV events was relatively small; and thirdly, the study duration of 52 weeks is short compared with studies dedicated to investigating CV outcomes.

In the IMPACT study, FF/UMEC/VI significantly reduced the rate of severe exacerbations compared with both dual therapies [22]. A post hoc analysis of the SUMMIT (NCT01313676) study data has shown that COPD exacerbations can increase the risk of CV events [9], and other studies have shown that CV involvement during a COPD exacerbation may contribute to poor outcomes [33, 34]. The greater reduction in the rate and risk of exacerbations observed with FF/UMEC/VI compared with either dual therapy in the IMPACT trial [22] may therefore be expected to reduce the risk of CV mortality. The IMPACT trial demonstrated a significant 28% reduction in on−/off-treatment all-cause mortality with FF/UMEC/VI versus UMEC/VI and a non-statistically significant reduction of 11% versus FF/VI, although CV mortality has not been specifically assessed [35, 36]. As the current analysis is focused on all CVAESI and only includes on-treatment events, it would be of interest to further explore the relationship between CV events, COPD exacerbations, and mortality in IMPACT in future analyses.

## Conclusions

In a large symptomatic COPD population with a history of exacerbations and a high rate of CV disease/risk, the proportion of patients with on-treatment CVAESI and MACE was 10–11% and 1–3%, respectively, and the risk of on-treatment CVAESI was low, with no consistent patterns across triple and dual treatment groups. These results for FF/UMEC/VI show no additive CV risk with bronchodilator combinations and are consistent with a previous network meta-analysis [37] and with the extensive existing CV safety database for FF/VI, UMEC/VI, and UMEC.

## Supplementary information

**Additional file 1: Supplementary Table 1**. SMQs, sub-SMQs, and Preferred Terms for on-treatment CVAESI that were reported in the IMPACT study **Supplementary Table 2**. Summary of on-treatment CVAESI by baseline CV risk factors (ITT population)

## Data Availability

Anonymized individual participant data and study documents can be requested for further research from www.clinicalstudydatarequest.com.

## Abbreviations

AE: Adverse event
AESI: Adverse event of special interest
CNS: Central nervous system
COPD: Chronic pulmonary obstructive disease
CV: Cardiovascular
CVAESI: Cardiovascular adverse event of special interest
FEV_1_: Forced expiratory volume in 1 s
FF: Fluticasone furoate
ICS: Inhaled corticosteroids
IMPACT: InforMing the Pathway of COPD Treatment
ITT: Intent-to-treat
LABA: Long-acting β_2_-agonists
LAMA: Long-acting muscarinic antagonists
MACE: Major adverse cardiovascular event
MedDRA: Medical dictionary for regulatory activities
MI: Myocardial infarction
NYHA: New York Heart Association
SAE: Serious adverse event
SMQ: Standardized MedDRA Query
TIO: Tiotropium
TTF: Time to first
UMEC: Umeclidinium
VI: Vilanterol
