# Supplementary material for: Single-inhaler triple therapy fluticasone furoate/umeclidinium/vilanterol versus fluticasone furoate/vilanterol and umeclidinium/vilanterol in patients with COPD: results on cardiovascular safety from the IMPACT trial
Source: Respir Res. 2020 Jun 5;21:139. doi: 10.1186/s12931-020-01398-w (PMC7275457; doi:10.1186/s12931-020-01398-w)
Supplement: Supplementary file 1 — Additional file 1: Supplementary Table 1. SMQs, sub-SMQs, and Preferred Terms for on-treatment CVAESI that were reported in the IMPACT study Supplementary Table 2. Summary of on-treatment CVAESI by baseline CV risk factors (ITT population) [file 12931_2020_1398_MOESM1_ESM.docx]

**SUPPLEMENTARY MATERIAL**

**Supplementary Table 1. SMQs, sub-SMQs, and Preferred Terms for on-treatment CVAESI that were reported in the IMPACT study**

| **AESI subgroup** | **Sub-SMQ** | **Preferred Term^1^** |
| --- | --- | --- |
| Cardiac arrhythmia | Arrhythmia-related investigations, signs and symptoms (SMQ) | Bradycardia |
|  |  | Cardiac arrest |
|  |  | Cardio-respiratory arrest |
|  |  | Electrocardiogram abnormal |
|  |  | Electrocardiogram change |
|  |  | Electrocardiogram repolarization abnormality |
|  |  | Heart rate decreased |
|  |  | Heart rate increased |
|  |  | Loss of consciousness |
|  |  | Palpitations |
|  |  | Sudden cardiac death |
|  |  | Sudden death |
|  |  | Syncope |
|  |  | Tachycardia |
|  | Bradyarrhythmia terms, nonspecific (SMQ)**^1^** |  |
|  | Cardiac arrhythmia terms, nonspecific (SMQ) | Arrhythmia |
|  |  | Heart rate irregular |
|  | Conduction defects (SMQ) | Atrioventricular block |
|  |  | Atrioventricular block complete |
|  |  | Atrioventricular block first degree |
|  |  | Atrioventricular dissociation |
|  |  | Bundle branch block |
|  |  | Bundle branch block bilateral |
|  |  | Bundle branch block left |
|  |  | Bundle branch block right |
|  |  | Defect conduction intraventricular |
|  |  | Electrocardiogram QT prolonged |
|  |  | Electrocardiogram repolarization abnormality |
|  | Disorders of sinus node function (SMQ) | Sinus arrest |
|  |  | Sinus arrhythmia |
|  |  | Sinus bradycardia |
|  |  | Sinus node dysfunction |
|  | Supraventricular tachyarrhythmias (SMQ) | Arrhythmia supraventricular |
|  |  | Atrial fibrillation |
|  |  | Atrial flutter |
|  |  | Atrial tachycardia |
|  |  | Sinus tachycardia |
|  |  | Supraventricular extrasystoles |
|  |  | Supraventricular tachycardia |
|  | Tachyarrhythmia terms, nonspecific (SMQ) | Cardiac fibrillation |
|  |  | Extrasystoles |
|  |  | Tachyarrhythmia |
|  | Ventricular tachyarrhythmias (SMQ) | Cardiac fibrillation |
|  |  | Ventricular arrhythmia |
|  |  | Ventricular extrasystoles |
|  |  | Ventricular fibrillation |
|  |  | Ventricular tachycardia |
| Cardiac failure (SMQ) |  | Acute left ventricular failure |
|  |  | Acute pulmonary edema |
|  |  | Brain natriuretic peptide increased |
|  |  | Cardiac failure |
|  |  | Cardiac failure acute |
|  |  | Cardiac failure chronic |
|  |  | Cardiac failure congestive |
|  |  | Cardiogenic shock |
|  |  | Cardiomegaly |
|  |  | Cardiopulmonary failure |
|  |  | Cor pulmonale |
|  |  | Cor pulmonale chronic |
|  |  | Diastolic dysfunction |
|  |  | Edema |
|  |  | Edema peripheral |
|  |  | Left ventricular failure |
|  |  | Lower respiratory tract congestion |
|  |  | Nocturnal dyspnea |
|  |  | Peripheral swelling |
|  |  | Pulmonary congestion |
|  |  | Pulmonary edema |
|  |  | Right ventricular failure |
| CNS hemorrhages and cerebrovascular conditions (SMQ) |  | Amaurosis fugax |
|  |  | Aphasia |
|  |  | Brain stem ischemia |
|  |  | Brain stem stroke |
|  |  | Carotid arteriosclerosis |
|  |  | Carotid artery occlusion |
|  |  | Carotid artery stenosis |
|  |  | Cerebellar infarction |
|  |  | Cerebral hemorrhage |
|  |  | Cerebral infarction |
|  |  | Cerebral ischemia |
|  |  | Cerebrovascular accident |
|  |  | Cerebrovascular disorder |
|  |  | Cerebrovascular insufficiency |
|  |  | Dysarthria |
|  |  | Hemiparesis |
|  |  | Hemorrhage intracranial |
|  |  | Hypoxic-ischemic encephalopathy |
|  |  | Intracranial aneurysm |
|  |  | Ischemic stroke |
|  |  | Lacunar infarction |
|  |  | Post procedural stroke |
|  |  | Thalamic infarction |
|  |  | Transient ischemic attack |
|  |  | Vascular stent restenosis |
|  |  | Vertebrobasilar insufficiency |
| Hypertension (SMQ) |  | Accelerated hypertension |
|  |  | Blood pressure diastolic increased |
|  |  | Blood pressure fluctuation |
|  |  | Blood pressure increased |
|  |  | Diastolic hypertension |
|  |  | Essential hypertension |
|  |  | Hypertension |
|  |  | Hypertensive crisis |
|  |  | Hypertensive emergency |
|  |  | Hypertensive heart disease |
|  |  | Labile blood pressure |
|  |  | Systolic hypertension |
| Ischemic heart disease (SMQ) |  | Acute coronary syndrome |
|  |  | Acute myocardial infarction |
|  |  | Angina pectoris |
|  |  | Angina unstable |
|  |  | Arteriosclerosis coronary artery |
|  |  | Blood creatine phosphokinase increased |
|  |  | Coronary artery disease |
|  |  | Coronary artery insufficiency |
|  |  | Coronary artery occlusion |
|  |  | Coronary artery stenosis |
|  |  | Electrocardiogram ST elevation |
|  |  | Electrocardiogram ST segment depression |
|  |  | Electrocardiogram T wave abnormal |
|  |  | Electrocardiogram T wave inversion |
|  |  | Ischemic cardiomyopathy |
|  |  | Myocardial infarction |
|  |  | Myocardial ischemia |
|  |  | Prinzmetal angina |
|  |  | Stress cardiomyopathy |
|  |  | Troponin increased |
|  |  | Vascular stent restenosis |

**^1^**Preferred Terms are only reported for events that occurred in at least 1 patient in any treatment arm. CNS, central nervous system; CVAESI, cardiovascular adverse events of special interest; MedDRA, Medical Dictionary for Regulatory Activities; SMQ, Standardized MedDRA Query.

**Supplementary Table 2. Summary of on-treatment CVAESI by baseline CV risk factors (ITT population)**

| **Special interest group/subgroup** | **FF/UMEC/VI**  **(N=4151)** | | | | **FF/VI**  **(N=4134)** | | | | **UMEC/VI**  **(N=2070)** | | | |
| --- | --- | --- | --- | --- | --- | --- | --- | --- | --- | --- | --- | --- |
| **No CV risk factors** |  |  | |  | |  | |  | | |  |  |
| **Number of patients in subgroup** | 1365 | | | 1322 | | | | 656 | | | |  |
| **Total duration at risk (patient-years)** | 1238.7 | | | 1124.7 | | | | 555.5 | | | |  |
|  | **n (%)** | **Rate [#]** | | **n (%)** | | **Rate [#]** | | **n (%)** | | | **Rate [#]** |  |
| **CVAESI** | **96 (7)** | **89.6 [111]** | | **96 (7)** | | **107.6 [121]** | | **46 (7)** | | | **97.2 [54]** |  |
| Cardiac arrhythmia | 27 (2) | 25.0 [31] | | 35 (3) | | 32.9 [37] | | 18 (3) | | | 32.4 [18] |  |
| Arrhythmia-related investigations, signs and symptoms (SMQ) | 13 (<1) | 11.3 [14] | | 12 (<1) | | 11.6 [13] | | 9 (1) | | | 16.2 [9] |  |
| Bradyarrhythmia terms, nonspecific (SMQ) | 0 (0) | 0 [0] | | 0 (0) | | 0 [0] | | 0 (0) | | | 0 [0] |  |
| Cardiac arrhythmia terms, nonspecific (SMQ) | 1 (<1) | 0.8 [1] | | 3 (<1) | | 2.7 [3] | | 1 (<1) | | | 1.8 [1] |  |
| Conduction defects (SMQ) | 2 (<1) | 1.6 [2] | | 6 (<1) | | 5.3 [6] | | 4 (<1) | | | 7.2 [4] |  |
| Disorders of sinus node function (SMQ) | 0 (0) | 0 [0] | | 0 (0) | | 0 [0] | | 0 (0) | | | 0 [0] |  |
| Supraventricular tachyarrhythmias (SMQ) | 10 (<1) | 8.1 [10] | | 11 (<1) | | 9.8 [11] | | 2 (<1) | | | 3.6 [2] |  |
| Tachyarrhythmia terms, nonspecific (SMQ) | 0 (0) | 0 [0] | | 2 (<1) | | 1.8 [2] | | 0 (0) | | | 0 [0] |  |
| Ventricular tachyarrhythmias (SMQ) | 4 (<1) | 3.2 [4] | | 3 (<1) | | 2.7 [3] | | 2 (<1) | | | 3.6 [2] |  |
| Cardiac failure (SMQ) | 28 (2) | 23.4 [29] | | 23 (2) | | 24.0 [27] | | 10 (2) | | | 19.8 [11] |  |
| CNS hemorrhages and cerebrovascular conditions (SMQ) | 8 (<1) | 6.5 [8] | | 3 (<1) | | 2.7 [3] | | 1 (<1) | | | 1.8 [1] |  |
| Hypertension (SMQ) | 31 (2) | 25.0 [31] | | 41 (3) | | 39.1 [44] | | 15 (2) | | | 30.6 [17] |  |
| Ischemic heart disease (SMQ) | 12 (<1) | | 9.7 [12] | | 10 (<1) | | 8.9 [10] | | 7 (1) | 12.6 [7] | |  |
| **1 CV risk factor** |  | | | |  | | | |  |  | |  |
| **Number of patients in subgroup** | 1147 | | | | 1158 | | | | 580 | | |  |
| **Total duration at risk (patient-years)** | 1021.8 | | | | 971.4 | | | | 465.9 | | |  |
|  | **n (%)** | | **Rate [#]** | | **n (%)** | | **Rate [#]** | | **n (%)** | **Rate [#]** | |  |
| **CVAESI** | **99 (9)** | | **112.5 [115]** | | **109 (9)** | | **139.0 [135]** | | **61 (11)** | **158.8 [74]** | |  |
| Cardiac arrhythmia | 31 (3) | | 35.2 [36] | | 39 (3) | | 45.3 [44] | | 15 (3) | 40.8 [19] | |  |
| Arrhythmia-related investigations, signs and symptoms (SMQ) | 12 (1) | | 12.7 [13] | | 22 (2) | | 25.7 [25] | | 6 (1) | 17.2 [8] | |  |
| Bradyarrhythmia terms, nonspecific (SMQ) | 0 (0) | | 0 [0] | | 0 (0) | | 0 [0] | | 0 (0) | 0 [0] | |  |
| Cardiac arrhythmia terms, nonspecific (SMQ) | 2 (<1) | | 2.0 [2] | | 3 (<1) | | 3.1 [3] | | 0 (0) | 0 [0] | |  |
| Conduction defects (SMQ) | 4 (<1) | | 3.9 [4] | | 3 (<1) | | 3.1 [3] | | 4 (<1) | 8.6 [4] | |  |
| Disorders of sinus node function (SMQ) | 0 (0) | | 0 [0] | | 1 (<1) | | 1.0 [1] | | 0 (0) | 0 [0] | |  |
| Supraventricular tachyarrhythmias (SMQ) | 13 (1) | | 12.7 [13] | | 7 (<1) | | 7.2 [7] | | 6 (1) | 12.9 [6] | |  |
| Tachyarrhythmia terms, nonspecific (SMQ) | 1 (<1) | | 1.0 [1] | | 1 (<1) | | 1.0 [1] | | 0 (0) | 0 [0] | |  |
| Ventricular tachyarrhythmias (SMQ) | 3 (<1) | | 2.9 [3] | | 4 (<1) | | 4.1 [4] | | 1 (<1) | 2.1 [1] | |  |
| Cardiac failure (SMQ) | 29 (3) | | 29.4 [30] | | 34 (3) | | 37.1 [36] | | 20 (3) | 45.1 [21] | |  |
| CNS hemorrhages and cerebrovascular conditions (SMQ) | 7 (<1) | | 6.9 [7] | | 13 (1) | | 16.5 [16] | | 5 (<1) | 10.7 [5] | |  |
| Hypertension (SMQ) | 23 (2) | | 24.5 [25] | | 27 (2) | | 28.8 [28] | | 15 (3) | 34.3 [16] | |  |
| Ischemic heart disease (SMQ) | 16 (1) | | 16.6 [17] | | 11 (<1) | | 11.3 [11] | | 13 (2) | 27.9 [13] | |  |
| **2 CV risk factors** |  | |  | |  | |  | |  |  | |  |
| **Number of patients in subgroup** | 1639 | | | | 1654 | | | | 834 | | |  |
| **Total duration at risk (patient-years)** | 1454.3 | | | | 1361.8 | | | | 676.9 | | |  |
|  | **n (%)** | | **Rate [#]** | | **n (%)** | | **Rate [#]** | | **n (%)** | **Rate [#]** | |  |
| **CVAESI** | **255 (16)** | | **271.6 [395]** | | **225 (14)** | | **210.7 [287]** | | **117 (14)** | **229.0 [155]** | |  |
| Cardiac arrhythmia | 95 (6) | | 83.9 [122] | | 87 (5) | | 71.2 [97] | | 48 (6) | 73.9 [50] | |  |
| Arrhythmia-related investigations, signs and symptoms (SMQ) | 38 (2) | | 31.6 [46] | | 37 (2) | | 30.1 [41] | | 18 (2) | 26.6 [18] | |  |
| Bradyarrhythmia terms, nonspecific (SMQ) | 0 (0) | | 0 [0] | | 0 (0) | | 0 [0] | | 0 (0) | 0 [0] | |  |
| Cardiac arrhythmia terms, nonspecific (SMQ) | 4 (<1) | | 2.8 [4] | | 4 (<1) | | 2.9 [4] | | 5 (<1) | 7.4 [5] | |  |
| Conduction defects (SMQ) | 14 (<1) | | 10.3 [15] | | 7 (<1) | | 5.1 [7] | | 2 (<1) | 3.0 [2] | |  |
| Disorders of sinus node function (SMQ) | 3 (<1) | | 2.1 [3] | | 1 (<1) | | 0.7 [1] | | 0 (0) | 0 [0] | |  |
| Supraventricular tachyarrhythmias (SMQ) | 42 (3) | | 32.3 [47] | | 33 (2) | | 27.2 [37] | | 19 (2) | 29.5 [20] | |  |
| Tachyarrhythmia terms, nonspecific (SMQ) | 2 (<1) | | 1.4 [2] | | 1 (<1) | | 0.7 [1] | | 1 (<1) | 1.5 [1] | |  |
| Ventricular tachyarrhythmias (SMQ) | 6 (<1) | | 4.1 [6] | | 6 (<1) | | 4.4 [6] | | 4 (<1) | 5.9 [4] | |  |
| Cardiac failure (SMQ) | 81 (5) | | 68.1 [99] | | 69 [4] | | 62.4 [85] | | 38 (5) | 65.0 [44] | |  |
| CNS hemorrhages and cerebrovascular conditions (SMQ) | 26 (2) | | 20.6 [30] | | 12 (<1) | | 9.5 [13] | | 5 (<1) | 7.4 [5] | |  |
| Hypertension (SMQ) | 59 (4) | | 52.3 [76] | | 47 (3) | | 36.0 [49] | | 24 (3) | 36.9 [25] | |  |
| Ischemic heart disease (SMQ) | 52 (3) | | 46.8 [68] | | 36 (2) | | 31.6 [43] | | 27 (3) | 47.3 [32] | |  |

#, number of events. Rates are reported as number of events per 1000 patient-years, calculated as the number of events x 1000, divided by the total duration at risk.
CNS, central nervous system; CVAESI, cardiovascular adverse event of special interest; FF, fluticasone furoate; ITT, intent-to-treat; MedDRA, Medical Dictionary for Regulatory Activities; n, number of patients; SMQ, Standardized MedDRA Query; UMEC, umeclidinium; VI, vilanterol.
